# Giant proximal left anterior descending aneurysm causing multi-vessel myocardial ischaemia: the pressure is on—a case report

**DOI:** 10.1093/ehjcr/ytad550

**Published:** 2023-11-07

**Authors:** Alexander Rutherford, Badrinathan Chandrasekaran, Mario Petrou, Steve Ramcharitar

**Affiliations:** Registrar, Great Western Hospital, Marlborough Road, Swindon SN3 6BB, UK; Great Western Hospital, Marlborough Road, Swindon SN3 6BB, UK; Royal Brompton and Harefield Hospitals, Sydney Street, London SW3 6NP, UK; Great Western Hospital, Marlborough Road, Swindon SN3 6BB, UK

**Keywords:** Case report, Coronary artery aneurysm, CT-FFR, Stress echocardiography, Coronary artery bypass graft

## Abstract

**Background:**

Giant coronary artery aneurysms are a rare cause of myocardial ischaemia. Due to the rarity and variety of presentation of these cases, no standardized investigation or treatment has been established for management. We report a case study of a giant proximal left anterior descending (LAD) coronary aneurysm causing myocardial ischaemia due to the pressure effect from the weight of the aneurysm as well as from a change in rheology from a ‘steal effect’ on both the LAD and left circumflex (LCx) arteries.

**Case summary:**

A 55-year-old patient presents initially with a history of angina. Initial investigation with computed tomography (CT) was suboptimal, requiring invasive diagnostic angiography, which detects a giant proximal LAD aneurysm. Subsequent investigations, with CT-fractional flow reserve (FFR) and stress echocardiography (ECHO), correlated to identify multi-vessel ischaemia resulting from the aneurysm. The patient was managed with multi-disciplinary team–led surgical resection and triple coronary artery bypass grafts with good results.

**Discussion:**

This case highlights the complexity of coronary anomalies and importance of additional functional three-dimensional imaging on top of the static computational tomography coronary angiography analysis. Together, these two complimentary investigations qualitatively enabled the assessment of anomaly with surrounding structures such that the possibility of a mass effect on the LCx artery results in a positive stress test. Furthermore, this is a novel use of CT-FFR for coronary anomalies and it demonstrated good correlation of LAD territory ischaemia between CT-FFR and the stress ECHO.

**Learning points**

- The management of coronary artery aneurysms benefits from multi-modality imaging both at rest and with stress imaging.
- In cases of coronary anomalies, computed tomography (CT)-fractional flow reserve (FFR) can correlate with stress echo findings.
- There is no consensus on medical management of coronary aneurysms given the diversity of presentations. Here, dual-antiplatelet therapy was safe prior to surgical resection and bypass grafting.

## Introduction

Aneurysmal coronary artery disease is a rare condition causing localized dilatation of the coronary artery by at least 50%, with population studies showing incidence of 0.3–5.3%.^1,2^ Coronary artery aneurysms (CAA) are predominantly right-sided, with a number of different causative factors including atherosclerosis, Takayasu’s arteritis, connective tissue disorders, infection, congenital and lesser so complications from percutaneous coronary intervention, coronary bypass surgery, and cocaine use.^1–3^ Giant CAA (>2 cm) are even rarer, with a reported incidence of 0.02–0.2%, and have a higher degree of complications including ischaemia, thrombosis, and rupture.^4,5^ Due to their rarity, there is no defined consensus on optimal management of CAA, from conservative with antiplatelets to surgical revascularization.^4,6^ Herein, we present a case report of a patient who presented with angina symptoms from a giant proximal left anterior descending (LAD) aneurysm with additional mass effect and inherent change in flow dynamics on both the LAD and the left circumflex (LCx) arteries.

## Summary figure

**Figure:**
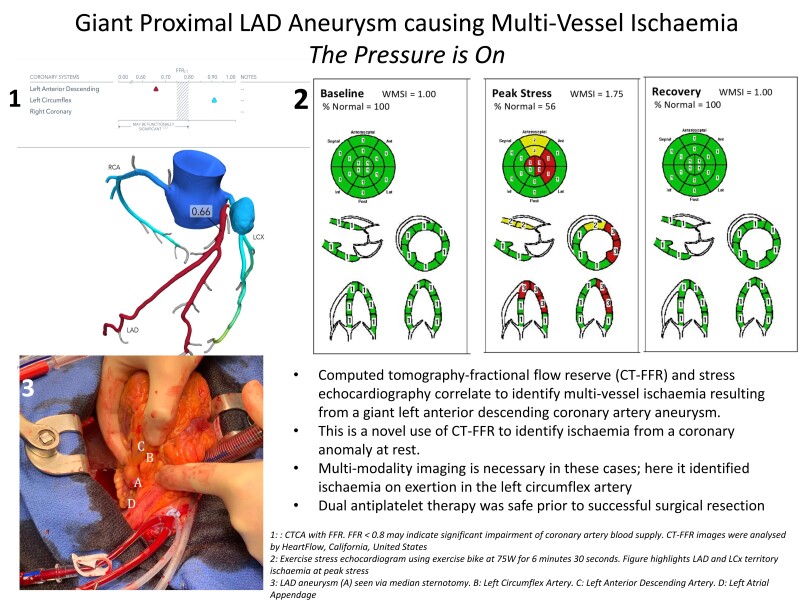


## Case summary

A 55-year-old female presented with a 4-month history of classical anginal symptoms to the nurse-led rapid access chest pain clinic. Past medical history included chronic obstructive pulmonary disease. The patient’s cardiac risk factors were that of being a current smoker with a positive family history of myocardial infarction. Initial assessment of her coronary arteries with coronary computed tomography angiogram (CCTA) was suboptimal due to patient motion artefact, a known limitation of this imaging modality. The motion artefact likely originated from an early ectopic beat or sinus arrhythmia. From the sections of coronary artery that were clearly visible, there was no coronary artery disease detected. The patient was referred to the general cardiology clinic to decide on further investigation and management. Despite her symptoms, medical management was not initiated at this stage as there was no known coronary artery disease. Subsequent follow-up occurred in clinic 2 months after her initial presentation, where there had been a progression of her anginal symptoms. Her chest pain was now occurring at rest and was much more pronounced on exertion.

There were no additional symptoms of palpitations, breathlessness, dizziness, or syncope. Electrocardiogram (ECG) showed normal sinus rhythm with no ischaemic changes. Cardiovascular examination was normal. The serum cholesterol was raised at 5.58, with the remainder of the lipid profile within the normal range. The blood count, renal function, electrolytes, and haemoglobin A1C (HbA1c) were all normal. The decision was made to proceed with invasive coronary angiography. The patient was started on aspirin, atorvastatin, and bisoprolol 2.5 mg once a day, which improved her symptoms.

**Figure 1:**
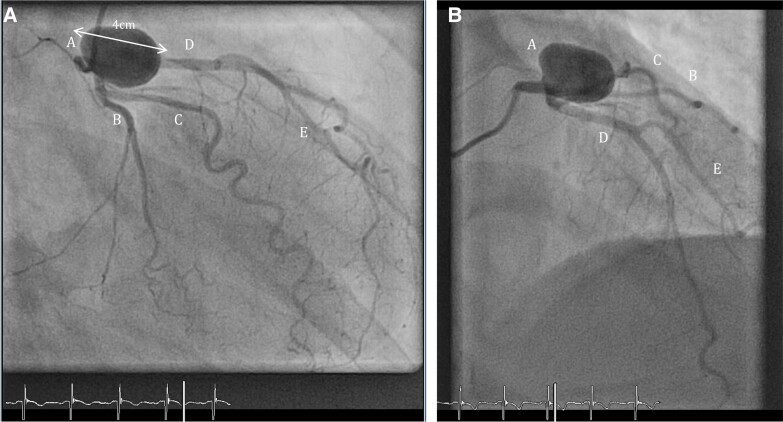
(*A*) Proximal left anterior descending (LAD) coronary artery aneurysm (CAA) displayed at angiography in cranial view: A, LAD aneurysm measuring 4 cm; B, left circumflex (LCx) artery; C, first obtuse marginal artery; D, LAD artery; E, first diagonal artery. (*B*) Proximal LAD coronary artery aneurysm displayed at angiography in caudal view: A, LAD aneurysm measuring 4 cm; B, LCx artery; C, first obtuse marginal artery; D, LAD artery; E, first diagonal artery.

Coronary angiogram via right radial artery access identified a large proximal saccular LAD aneurysm (∼4 cm in diameter) without evidence of thrombus (*Figure 1*).

The patient was discussed at the cardiothoracic multi-disciplinary team (MDT) meeting, with decision to proceed with a further CCTA with CT-fractional flow reserve (CT-FFR) pressure studies and stress echocardiography (ECHO) to identify if the LAD aneurysm was having a significant ischaemic effect at rest but also if ischaemia worsened with activity. On the initial CT coronary angiogram, where motion artefact limited identification and diagnosis of this aneurysm, the aneurysm was visible when assessed retrospectively and missed by the initial CCTA reader. Furthermore, due to the initial image quality, there was no visualization of the connection of the aneurysm to the cardiac anatomy (see Supplementary material online, *Video S1*). The decision to repeat the CCTA was due to the three-dimensional (3D) view offered by CT that assists in surgical planning and additionally providing a non-invasive measure of FFR; which would have been high risk to achieve via invasive angiography. Due to the patient having a resting heart rate of 55 b.p.m., the same CT protocol (prospective gating in diastole without wire padding) was used to obtain the images.

**Figure 2:**
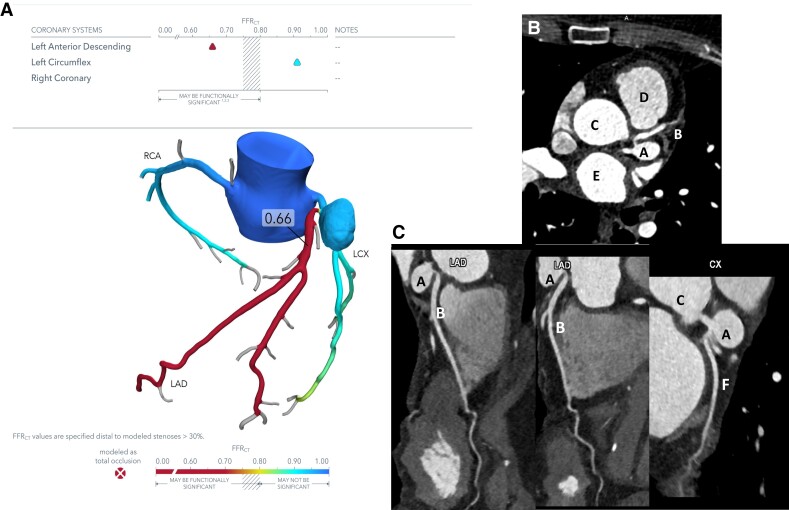
(*A*) CTCA with FFR. FFR < 0.8 may indicate significant impairment of coronary artery blood supply. CT-FFR images were analysed by HeartFlow, California, USA. (*B*) Coronary CT angiogram axial view. (*C*) Coronary CT angiogram, variable views assessing aneurysmal anatomy in relation to left anterior descending or left circumflex artery. A, coronary aneurysm; B, left anterior descending artery; C, ascending aorta; D, right ventricle; E, left atrium; F, left circumflex artery.

Coronary computed tomography angiogram found the aneurysm to be smaller than suspected in angiography but still significant, measuring 2 cm in diameter with a small neck. There was significant impairment of coronary artery blood supply in the LAD artery (*Figure 2* and Supplementary material online, *Videos S2*​and*S3*). There was no evidence of impingement or impairment of coronary artery blood supply on the LCx artery at rest. The patient was started on dual-antiplatelet therapy to reduce the risk of thrombosis.^6^

**Figure 3:**
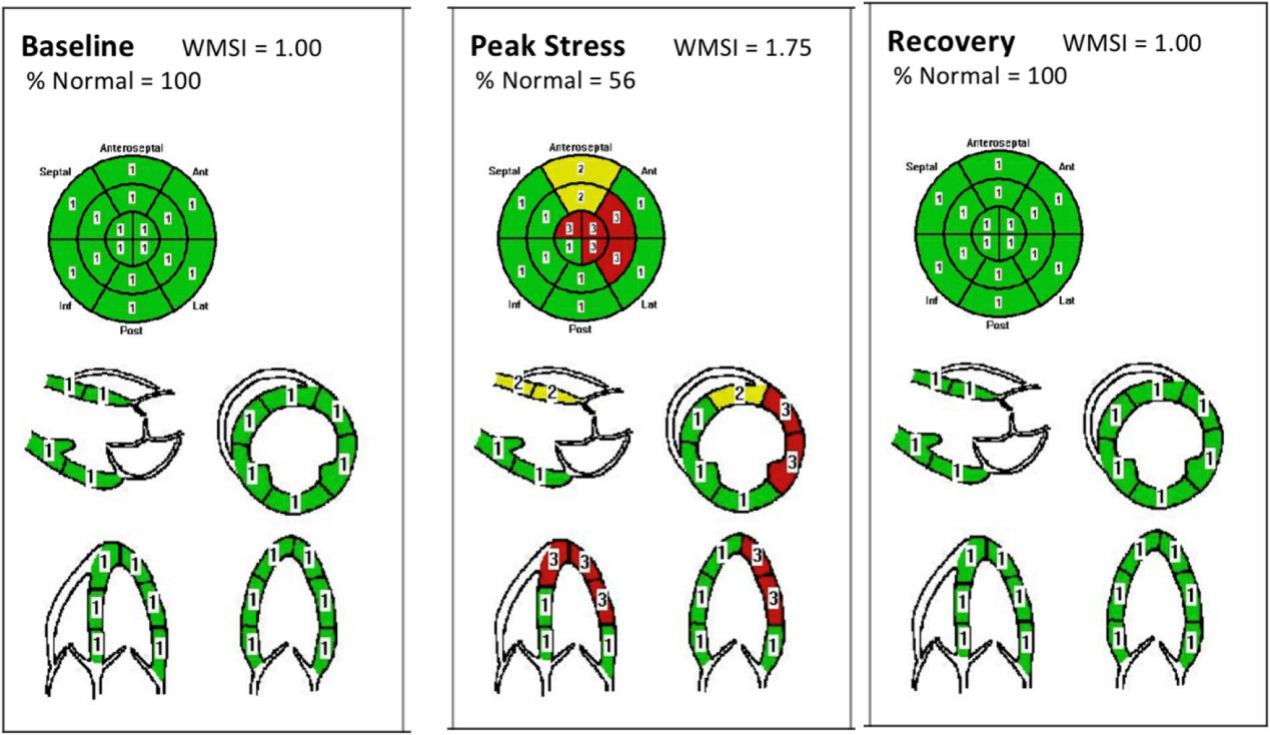
Exercise stress ECHO using exercise bike at 75 W for 6 min 30 s. Figure highlights left anterior descending and left circumflex territory ischaemia at peak stress. Territories marked [1] display areas with normal wall motion. Territories marked [2] are hypokinetic. Territories marked [3] are akinetic.

Exercise stress ECHO was performed on an exercise bike for 6 min and 30 s. Heart rate varied from 65 to 146 b.p.m. (>85% of target heart rate). At peak stress, the stress ECHO unequivocally showed LAD territory ischaemia, as well as to a lesser extent LCx territory ischaemia (*Figure 3* and Supplementary material online, *Video S2*). At peak stress, the mid- and apical anterolateral walls were akinetic, while the basal and mid-anteroseptum were hypokinetic. Ejection fraction reduced from 69 to 50% at peak stress indicative of ischaemia. The patient was asymptomatic during the stress ECHO, possibly as a result of the prior initiation of optimal medical therapy.

**Figure 4:**
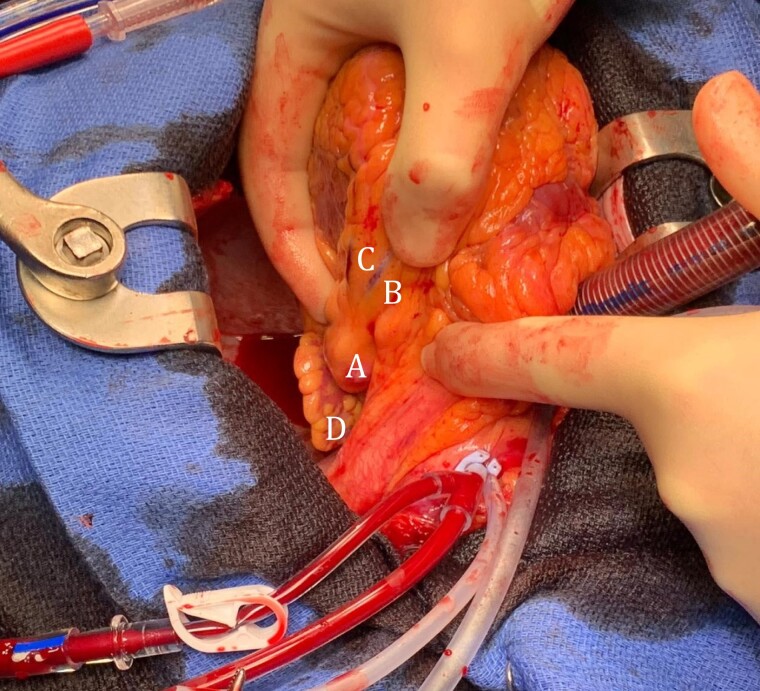
Left anterior descending (LAD) aneurysm (A) seen via median sternotomy. B, Left circumflex artery; C, left anterior descending artery; D, left atrial appendage.

The large giant CAA seen on angiography correlated well with LAD territory ischaemia seen at rest on CT-FFR. Furthermore, on exertion, there was evidence of LAD territory ischaemia as well as LCx territory ischaemia seen on exertion on stress ECHO. This was likely due to external compression of the LAD and LCx arteries during exercise in the rather confined anatomical space. Discussion at MDT supported an operative management. Left internal mammary artery (LIMA) and segments of the left and right great saphenous vein grafts (SVG) were harvested synchronously. On opening via median sternotomy, the left coronary aneurysm was easily seen posterior to the main pulmonary artery and anterior to the base of the left atrial appendage (*Figure 4*).

**Figure 5:**
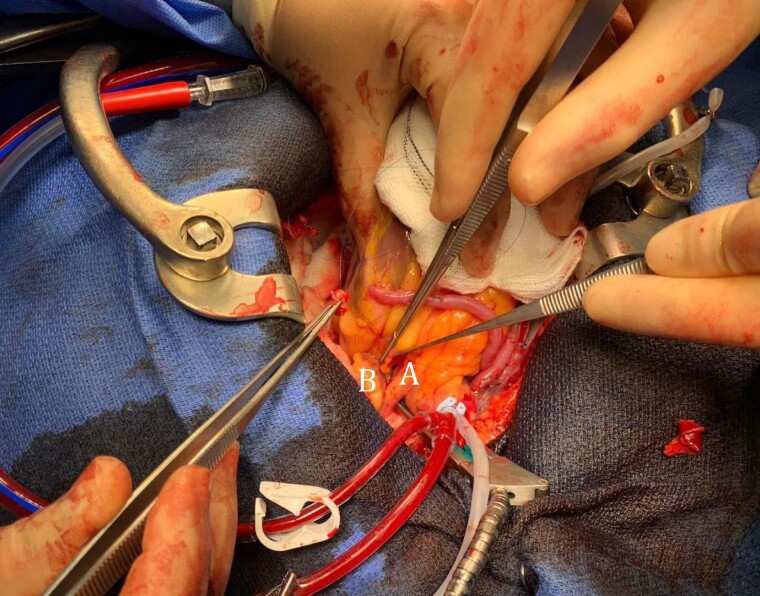
Transfixion of the left anterior descending aneurysm (A). B, Left atrial appendage.

Following full systemic heparinization, cardiopulmonary bypass was instituted and the patient cooled to 32°C. The aortic cross clamp was applied and 1200 mL of cardioplegia was administered. The middle and proximal LAD segments were intramuscular, reducing the ability to bypass the aneurysm proximally. The patient had LIMA to diagonal-1, SVG to LAD, and SVG to obtuse marginal-1 grafts successfully constructed. The coronary aneurysm sac was devoid of any obvious thrombus and was not calcified; therefore, the decision was made to transfix the base with continuous 5/0 Prolene to exclude it from the circulation and buttressed with autologous pericardium (*Figure 5*). The two proximal anastomoses were then constructed with continuous 5/0 Prolene. After rewarming, the patient was successfully weaned and disconnected from cardiopulmonary bypass with no ionotropic support and paced AAI at 90 b.p.m. overnight.

### Follow-up

The patient was discharged following an uncomplicated stay. The last follow-up was performed at 13 months following the procedure, and the patient has remained free of any complications. A follow-up ECHO performed at 6 months demonstrated normal biventricular size and function.

## Discussion

Diagnosis is difficult in patients with CAAs as there are no characteristic clinical features. This case adds to the body of evidence regarding the pathophysiology and presentation of CAAs, in particular highlighting that ischaemia can result from a combination of abnormal flow as well as extrinsic compression effecting two different coronary artery territories. There is limited information on adult patients with CAA without concurrent atherosclerotic coronary disease. A large review illustrated that patients with CAAs were more likely to have three-vessel disease, have a history of myocardial infarction, and be male, and 98% had concurrent coronary artery stenosis.^7^ A potential explanation of the low rate of CAAs found *in vivo* in the absence of coronary disease may be fatal rupture making it important to identify at-risk individuals.

Interestingly, her first presentation with chest pains was 4 months preceding the patient’s angiographic work-up. A missed ischaemic event potentially could have been the aetiology for the akinaesia seen on her subsequent stress ECHO. Coronary computed tomography angiogram is by far the gold standard to identify and monitor progression of these anomalies, but in this case, it failed to do so.^7–9^ The inherent limitation of motion artefact in CCTA did limit image quality. However, retrospective assessment of the initial CCTA indicated that the coronary aneurysm was visible in this scan and was missed. This further illustrates the limitations of the imaging modality through the importance of the CT readers’ understanding of and experience with coronary lesions, coronary anomalies, and anatomical variants. Furthermore, it highlights the potential complexity of these scans and the requirement to assess the CCTA results in conjunction with the patient’s clinical presentation. Unlike CCTA, invasive coronary angiography has the limitation of not offering a 3D view of the pathology, which can be important when planning surgery and in appreciating the spatial arrangement of coronary lesions. Care must be taken with invasive procedures such as angiography, and there is an increasing role for non-invasive imaging to assess ischaemic burden; however, in this case, it was a key step in the diagnostic pathway. There is growing data to support the role of CT-FFR in determining ischaemia in coronary arteries, but there is no randomized data or series assessing this novel technology to coronary anomalies.^10^ Our case demonstrates that there is a degree of correlation of ischaemia seen in CT-FFR with the stress ECHO findings in the LAD likely as a consequence of the change in rheology associated with a so-called steal effect at rest but also that the stress ECHO demonstrates further ischaemia on exertion in the territory of the LCx artery. This is likely as a result of mass effect compression within the confined cardiac space on exertion alone. Coronary computed tomography angiogram, being indicative of coronary flow at rest did not detect this. This case demonstrates the benefits of multi-modality imaging of coronary anomalies.

## Conclusion

Management of giant CAA is varied and multi-factorial, given the diversity of aetiology and presentation. Consideration of size, location, and concurrent atherosclerosis also alters management.^1,6^ In this case, dual-antiplatelet therapy sufficiently protected against the risk of thrombosis without leading to rupture of the aneurysm. Due to the location and mass effect causing two-vessel coronary artery ischaemia, surgical management was decided upon. Previous surgical management of CAA includes resection with end-to-end ligation, reconstruction, ligation plus coronary artery bypass, and aneurysmectomy.^1,6,7,11^

## Supplementary Material

ytad550_Supplementary_Data

## Data Availability

The data underlying this article are available in the article and in its online Supplementary material.

## Lead author biography

Alexander J. Rutherford is a Cardiology Registrar in Great Western Hospital, Swindon. He completed his Bachelor of Medicine and Bachelor of Surgery at Cardiff University, also completing his BSc in Medical Genetics with first class honours. He has a clinical interest in cardiology and wishes to pursue a career as a cardiologist.

## Supplementary material

**Consent:** The authors confirm that written consent for submission of the case report including images and associated text has been obtained from the patient in line with the Committee on Publication Ethics (COPE) guidance.

**Funding:** None declared.
