# Supplementary material for: Giant proximal left anterior descending aneurysm causing multi-vessel myocardial ischaemia: the pressure is on—a case report
Source: Eur Heart J Case Rep. 2023 Nov 7;7(11):ytad550. doi: 10.1093/ehjcr/ytad550 (PMC10665038; doi:10.1093/ehjcr/ytad550)
Supplement: ytad550_Supplementary_Data [file ytad550_supplementary_data.zip › Supplementary Video 1.pptx]

## Slide 1
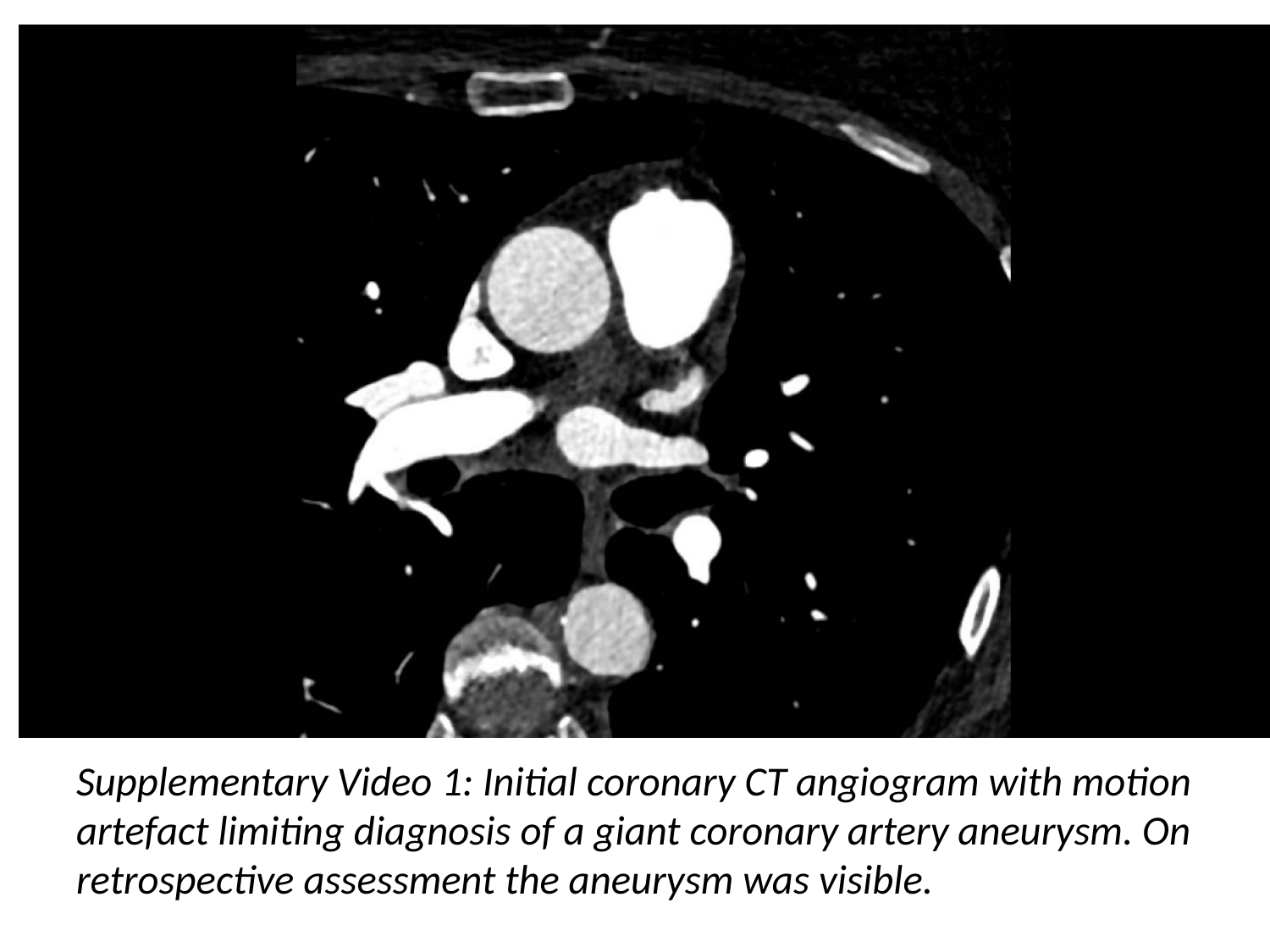

Supplementary Video 1: Initial coronary CT angiogram with motion artefact limiting diagnosis of a giant coronary artery aneurysm. On retrospective assessment the aneurysm was visible.
