# Supplementary material for: Giant proximal left anterior descending aneurysm causing multi-vessel myocardial ischaemia: the pressure is on—a case report
Source: Eur Heart J Case Rep. 2023 Nov 7;7(11):ytad550. doi: 10.1093/ehjcr/ytad550 (PMC10665038; doi:10.1093/ehjcr/ytad550)
Supplement: ytad550_Supplementary_Data [file ytad550_supplementary_data.zip › Supplementary Videos 2 and 3.pptx]

## Slide 1
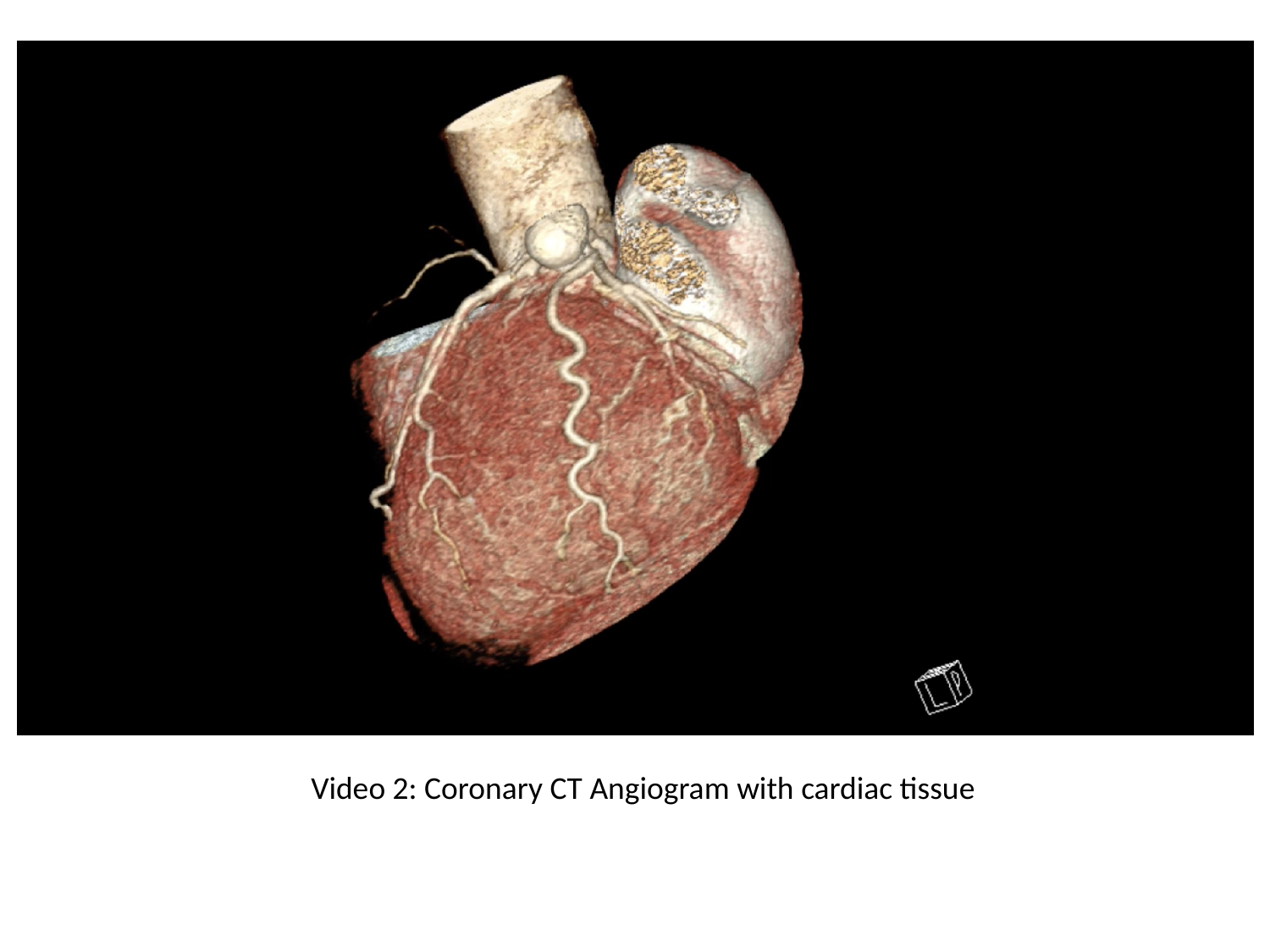

Video 2: Coronary CT Angiogram with cardiac tissue

## Slide 2
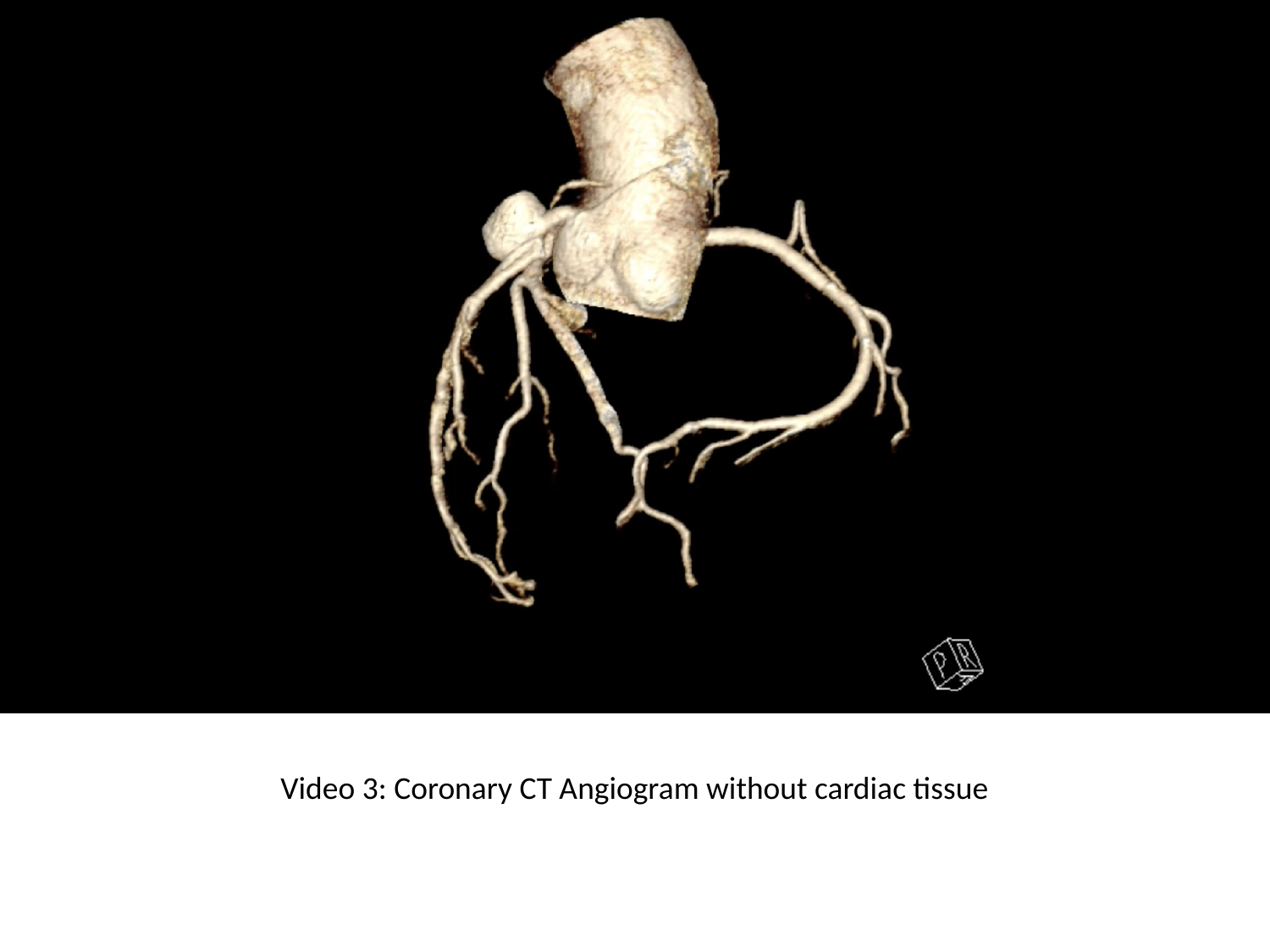

Video 3: Coronary CT Angiogram without cardiac tissue
