# Supplementary material for: Giant proximal left anterior descending aneurysm causing multi-vessel myocardial ischaemia: the pressure is on—a case report
Source: Eur Heart J Case Rep. 2023 Nov 7;7(11):ytad550. doi: 10.1093/ehjcr/ytad550 (PMC10665038; doi:10.1093/ehjcr/ytad550)
Supplement: ytad550_Supplementary_Data [file ytad550_supplementary_data.zip › Supplementary images LAD aneurysm 2.pptx]

## Slide 1
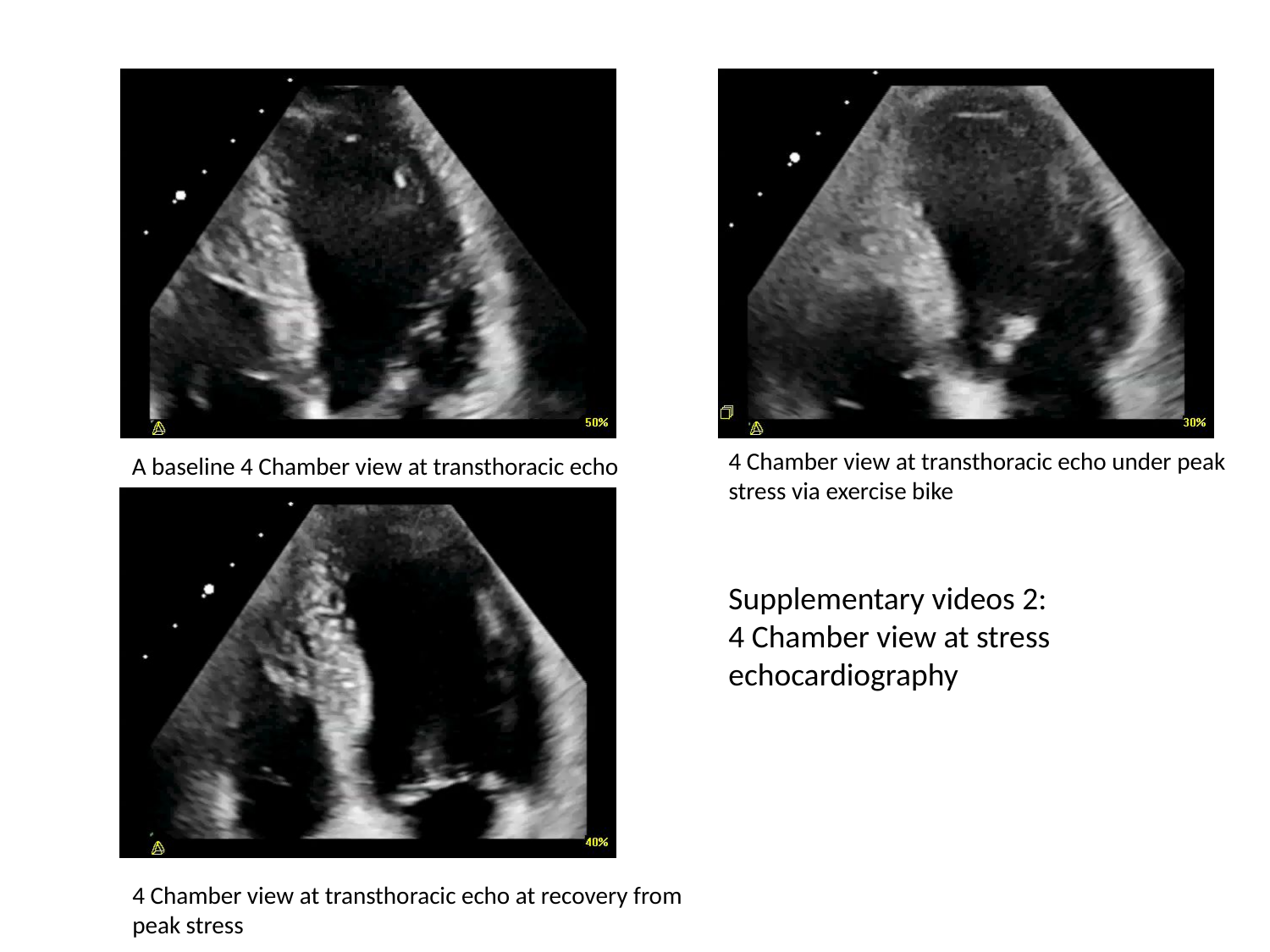

4 Chamber view at transthoracic echo under peak stress via exercise bike
A baseline 4 Chamber view at transthoracic echo
Supplementary videos 2:
4 Chamber view at stress echocardiography
4 Chamber view at transthoracic echo at recovery from peak stress

## Slide 2
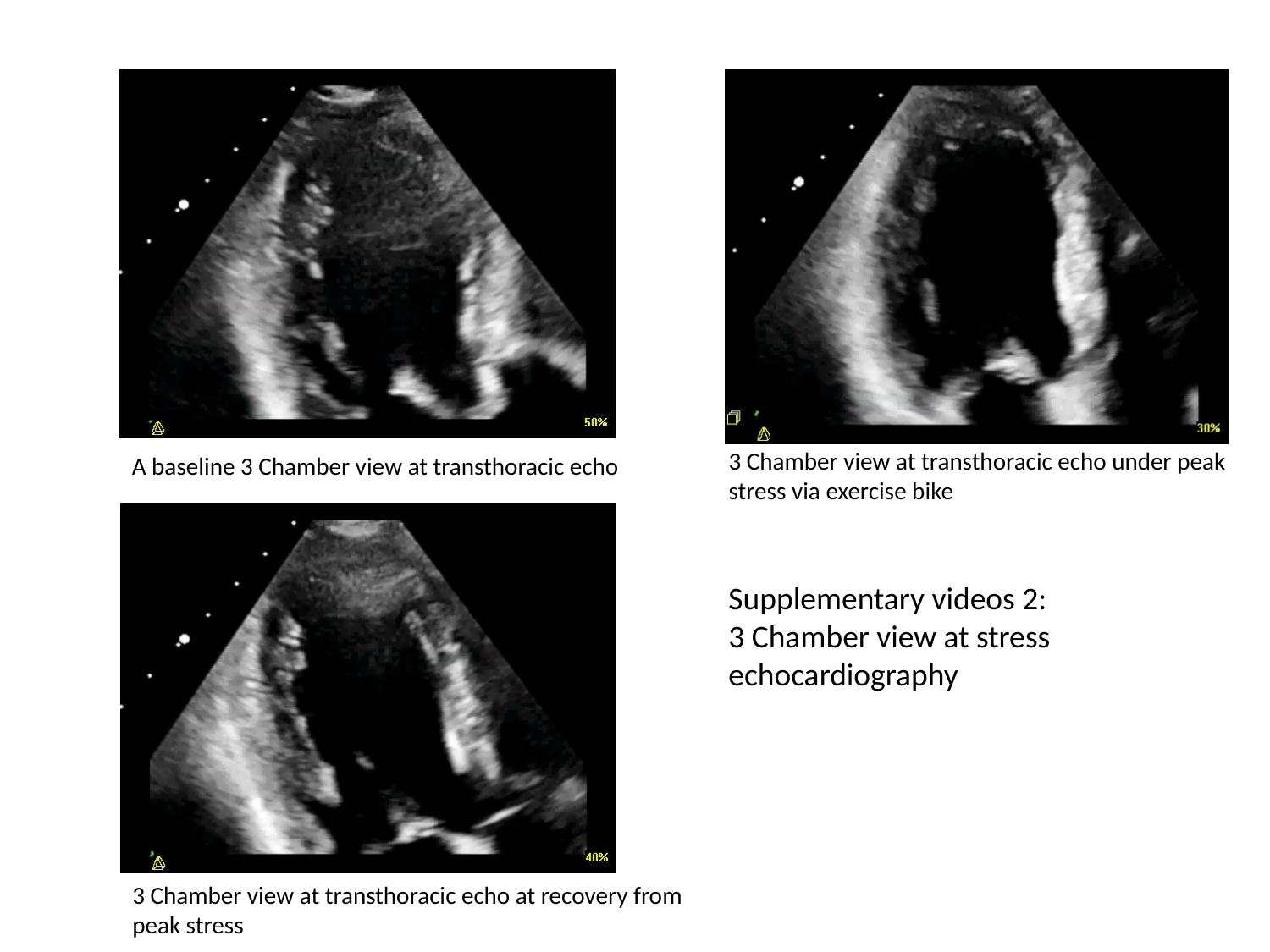

3 Chamber view at transthoracic echo under peak stress via exercise bike
A baseline 3 Chamber view at transthoracic echo
Supplementary videos 2:
3 Chamber view at stress echocardiography
3 Chamber view at transthoracic echo at recovery from peak stress

## Slide 3
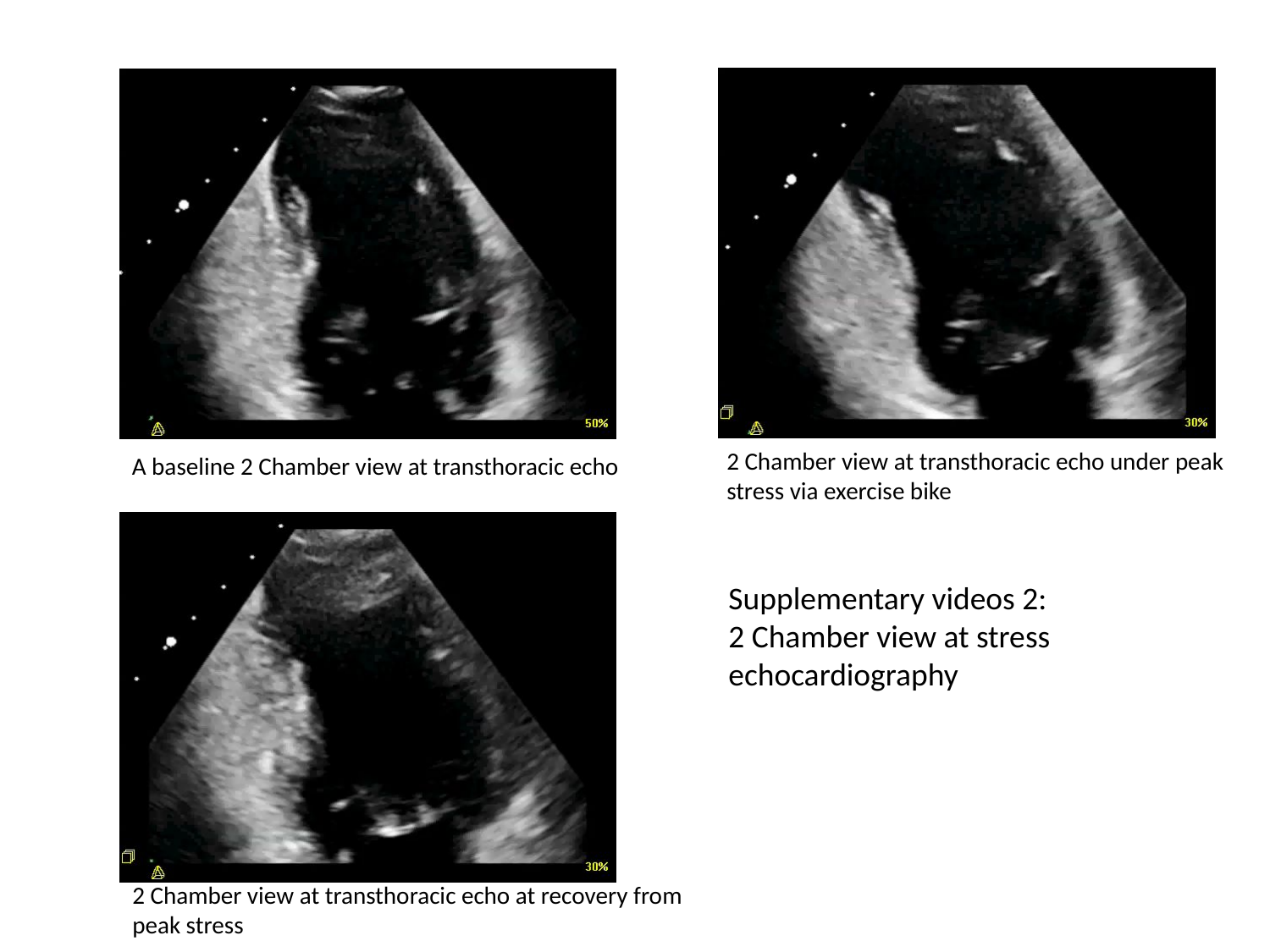

2 Chamber view at transthoracic echo under peak stress via exercise bike
A baseline 2 Chamber view at transthoracic echo
Supplementary videos 2:
2 Chamber view at stress echocardiography
2 Chamber view at transthoracic echo at recovery from peak stress
